# Proteomics Analysis to Assess the Role of Mitochondria in BRCA1-Mediated Breast Tumorigenesis

**DOI:** 10.3390/proteomes6020016

**Published:** 2018-03-27

**Authors:** Antonio Concolino, Erika Olivo, Laura Tammè, Claudia Vincenza Fiumara, Maria Teresa De Angelis, Barbara Quaresima, Valter Agosti, Francesco Saverio Costanzo, Giovanni Cuda, Domenica Scumaci

**Affiliations:** 1 Laboratory of Proteomics, Research Center of Advanced Biochemistry and Molecular Biology, Department of Experimental and Clinical Medicine, Magna Græcia University of Catanzaro, 88100 Catanzaro, Italy; a.concolino@unicz.it (A.C.); erika.olivo26@gmail.com (E.O.); tamme@unicz.it (L.T.); claudia.fiumara@live.it (C.V.F.); cuda@unicz.it (G.C.); 2 Stem Cell Laboratory, Research Center of Advanced Biochemistry and Molecular Biology, Department of Experimental and Clinical Medicine, Magna Graecia University of Catanzaro, Salvatore Venuta University Campus, 88100 Catanzaro, Italy; mariateresadeangelis211285@gmail.com; 3 Laboratory of Molecular Oncology, Department of Experimental and Clinical Medicine, Magna Graecia University, 88100 Catanzaro, Italy; quaresi@unicz.it (B.Q.); agosti@unicz.it (V.A.); Fsc@unicz.it (F.S.C.); 4 CIS for Genomics and Molecular Pathology, Magna Graecia University of Catanzaro, 88100 Catanzaro, Italy

**Keywords:** mitochondria, HIF-1α, 2D-DIGE, breast cancer, *BRCA1*

## Abstract

Mitochondria are the organelles deputed to energy production, but they are also involved in carcinogenesis, cancer progression, and metastasis, playing a role in altered energy metabolism in cancer cells. Mitochondrial metabolism is connected with several mitochondrial pathways such as ROS signaling, Ca^2+^ homeostasis, mitophagy, and mitochondrial biogenesis. These pathways are merged in an interactive super-network that seems to play a crucial role in cancer. Germline mutations of the *BRCA1* gene account for 5–10% of breast cancers and confer a risk of developing the disease 10- to 20-fold much higher than in non-carriers. By considering metabolic networks that could reconcile both genetic and non-genetic causal mechanisms in *BRCA1* driven tumorigenesis, we herein based our study on the hypothesis that *BRCA1* haploinsufficiency might drive metabolic rewiring in breast epithelial cells, acting as a push toward malignant transformation. Using 2D-DIGE we analyzed and compared the mitochondrial proteomic profile of sporadic breast cancer cell line (MCF7) and *BRCA1* mutated breast cancer cell line (HCC1937). Image analysis was carried out with Decider Software, and proteins differentially expressed were identified by LC-MS/MS on a quadrupole-orbitrap mass spectrometer Q-Exactive. Ingenuity pathways analysis software was used to analyze the fifty-three mitochondrial proteins whose expression resulted significantly altered in response to *BRCA1* mutation status. Mitochondrial Dysfunction and oxidative phosphorylation, and energy production and nucleic acid metabolism were, respectively, the canonical pathway and the molecular function mainly affected. Western blotting analysis was done to validate the expression and the peculiar mitochondrial compartmentalization of specific proteins such us HSP60 and HIF-1α. Particularly intriguing is the correlation between *BRCA1* mutation status and HIF-1α localization into the mitochondria in a *BRCA1* dependent manner. Data obtained led us to hypothesize an interesting connection between *BRCA1* and mitochondria pathways, capable to trigger metabolic changes, which, in turn, sustain the high energetic and anabolic requirements of the malignant phenotype.

## 1. Introduction

Worldwide, breast cancer (BC) is the most commonly diagnosed cancer type and represents the leading cause of cancer death among women. It is genetically and morphologically highly heterogeneous and subgrouping relying on pathological and clinical data can only partially reflect the clinical diversity of the disease.

Generally, breast cancer is associated with somatic mutations in breast cells acquired during a women’s lifetime. 5–10 percent of all cases are hereditary breast cancer, and are associated with distinct mutations on specific genes such us *BRCA1*, *BRCA2*, *PTEN* and *CHEK2. BRCA1* mutation carriers seem to have specific pathological features and gene expression profile [1,2].

*BRCA1* gene has been extensively studied, more than 1600 mutations have been described and the majority of them are frameshifts mutations resulting in the deletion or non-functional protein.

*BRCA1* is implicated in DNA double-strand break repair, transcriptional regulation, cell cycle control, apoptosis and resistance to chemotherapy [3].

BRCA1 is known to interact with several cellular players, including p53, c-Myc, AKT and HIF-1α [4,5].

The comprehension of the functional significance of hereditary mutations may improve breast cancer prevention and the assessment of promising strategies of treatment.

In *BRCA1*-driven tumorigenesis, in contrast with the two-hit theory [6], the haploinsufficiency status produces genomic instability in breast epithelial cells and acts as a push toward malignant transformation. Several evidences suggest that the inactivation of a single *BRCA1* allele induces a huge number of genetic alterations and the consequent acquisition of “permissive” status that leads to the homozygous loss of *BRCA1* [7,8,9].

The Warburg effect represents a major metabolic hallmark of cancer cells. The aerobic glycolysis occurs in normal cells during hypoxia and in cancer cells all the time. Hypoxia inducible factor-1α (HIF-1α) is the key regulator of the hypoxia response. In presence of low oxygen in the cell, pyruvate is converted to lactate instead of acetyl CoA, HIF-1α does not undergo proteasomal degradation and translocates into the nucleus, where it regulates the expression of genes involved in glucose metabolism. During hypoxia, normal cells undergo apoptosis, while cancer cells adapt to the low oxygen environment and survive [10,11].

The role of *BRCA1* in cancer cell metabolism remains to be elucidated, although very recently it has been shown that *BRCA1* haploinsufficiency regulates the oxidative mitochondrial metabolism and constitutes an early and important “hit” that drives the tumorigenesis process [12,13].

Mitochondria are the powerhouse of all living cells; they have a key role in several pathways such as apoptosis, Ca^2+^ homeostasis, ROS signaling and mitophagy. They are also involved in carcinogenesis, cancer progression and metastasis, playing a role in altered energy metabolism of cancer cells.

In this work, in order to characterize the main role of *BRCA1*-induced metabolic reprogramming, we performed DIGE experiments coupled with LC_MS/MS analysis to profile the mitochondria proteome of breast cancer cells expressing or not *BRCA1*.

The object of our study was to shed light on potential contribution of *BRCA1* to the metabolic features of cancer cells, including the so-called ‘‘Warburg effect’’.

## 2. Materials and Methods

### 2.1. Cells Culture

MCF-10, MCF-7 and HCC1937 cell lines were purchased from the American Type Culture Collection (Rockville, MD, USA). Human breast cancer cell line MCF7 was grown in Dulbecco’s modified Eagle’s medium (DMEM) (Sigma Aldrich, Saint Louis, MO, USA) supplemented with 10% (*w*/*v*) fetal bovine serum (FBS) (Sigma Aldrich), 100 mg/mL streptomycin and 100 U/mL penicillin (Sigma Aldrich). MCF10 mammary epithelial cell line was grown in MEGM Mammary Epithelial Cell Growth Medium (Lonza, Walkersville, MD, USA) supplemented with 20 ng/mL epidermal growth factor (Lonza), 0.5 µg/mL hydrocortisone (Lonza), 100 ng/mL cholera toxin (Sigma Aldrich) and 10 µg/mL insulin (Lonza). HCC1937 cell line was homozygous for the *BRCA1* 5382C mutation and was used as a model of hereditary breast cancer. HCC1937 cells (ATCC) were grown in RPMI medium (ATCC) supplemented with 20% (*w*/*v*) fetal bovine serum (FBS) (Sigma Aldrich), 100 mg/mL streptomycin and 100 U/mL penicillin (Sigma Aldrich).

All cell lines were cultured at a constant temperature of 37 °C in a 5% carbon dioxide (CO_2_) humidified atmosphere.

### 2.2. Full-Length Transfection of BRCA1 in HCC1937 Breast Cancer Cells

HCC1937/*^wtBRCA1^* cells were generated in our laboratory by full-length *BRCA1* cDNA transfection [14,15].

Parental HCC1937 were transfected with a pcDNA 3.1 plasmid containing the full-length *BRCA1* gene. Cells between 30–40% confluency were incubated overnight with 2 mg of plasmid DNA, using the FuGENE 6 transfection reagent (Roche Molecular Biochemicals, Monza, Italy) according to the manufacturer’s instructions. Stable transfectants were selected using G418 (0.4 mg/mL) (Invitrogen Life Technologies, La Jolla, CA, USA). HCC1937/*^wtBRCA1^* cells were amplified and used for further analysis

### 2.3. Whole Protein Extraction

Cells lines were washed with PBS and lysed with a buffer containing 15 mM Tris pH 7.5, 120 mM NaCl, 25 mM KCl, 0.5% Triton X-100, supplemented with protease and phosphatase inhibitor cocktail (Halt Protease Inhibitor Cocktail/Halt Phosphatase Inhibitor Cocktail, Thermo Fisher Scientific Inc., Bremen, Germany). Cell lysate was sonicated for 10 s and centrifuged at 15,000× *g* for 20 min. All the operations were performed at 4 °C. Supernatant was carefully removed and protein content was measured by the Bradford method (BioRad, Hercules, CA, USA); and the supernatants were stored at 80 °C.

### 2.4. Mitochondrial Fraction

Mitochondria were isolated according to previous literature [16,17,18]. Briefly, normal and cancer cells were washed with ice cold PBS, collected by gentle scrapping in cold PBS and centrifugalized at 600× *g*, at 4 °C for 10 min. Resulting pellet was suspended in 200 mM sucrose, 10 mM Tris–MOPS and 1 mM EDTA/Tris, pH 7.4 (STE buffer). Cells were homogenized by glass Potter homogenization and mitochondria were then isolated by serial centrifugations. Resulting supernatant was used as cytosolic fraction. The mitochondrial pellet was suspended in DIGE lysis buffer and protein content was assayed using the Bradford Protein Assay (Bio-Rad) according to the manufacturer’s instructions.

### 2.5. Isolation of Nuclear Fractions

Cells were collected with 1 mL of hypotonic lysis buffer (10 mM Tris-Cl pH 8.0, 1 mM KCl, 1.5 mM MgCl_2_, 1 mM DTT, supplemented with protease and phosphatase inhibitor cocktail) and incubated for 30 min on rotator at 4 °C. Cell lysate was centrifugated at 10,000× *g* for 10 min at 4 °C to isolate nuclei. Cytosol was supplemented with 1% Triton X-100. Both fractions were incubated on ice for 30 min and then centrifuged at 15,000× *g* for 20 min at 4 °C. Protein concentration was determined using the Bradford Protein Assay (Bio-Rad) according to the manufacturer’s instructions.

### 2.6. 2D-DIGE

Cell pellets were suspended in 7 M urea, 2 M tiourea, 4% chaps, 30 mM Tris pH 8.5, containing protease and Phosphatase inhibitor cocktail (Halt Protease Inhibitor Cocktail/Halt Phosphatase Inhibitor Cocktail, Thermo Fisher Scientific Inc., Bremen, Germany) and sonicated in an ultrasonic bath for 1 min. Proteins were quantified using the Bradford protein assay (Bio-Rad, Hercules, CA, USA), according to manufacturer’s instructions.

Protein extraction and solubilization were carried out in triplicate. Mitochondrial protein extraction was verified by western blot analysis.

2D-DIGE analysis was performed according to previously literature [19]. Mitochondrial proteins from cancer cells were labeled using the CyDyes™DIGE Fluors (Cy2, Cy5 and Cy3, GE Healthcare, Buckinghamshire, UK) at the ratio of 1 μg protein: 400 pmol dye.

Three biological replicates were prepared for each cellular model in order to perform a statistical significant experiment. Cy3 and Cy5 were used to label the samples, and Cy2 was used to label the internal standard, a pool of samples created by mixing an aliquot of all biological samples used for the analysis.

Protein labelling was carried out incubating each proteins extract with the respective cyanine for 30 min in the dark, at the pH of 8.5 (Table S1). Reactions were quenched adding 10 mM lysine, for 10 min in the dark.

Labeled extract were mixed and resuspended into Isoelectrofocusing (IEF) sample buffer containing 8 M urea, 4% CHAPS, 0.1 M DTT, 0.8% IPG buffer pH 3–10 NL. IEF was performed using non-linear precast IPG strip (pH 3–10 NL; 24-cm-long, GE Healthcare). The first dimension was carried out on GE Healthcare IPGphor unit, until a total of 70,000 Vh was reached. After the first dimension, IPG strips were equilibrated with SDS equilibration solution containing 10 mg/mL dithiothreitol, followed by a treatment with SDS equilibration solution containing 25 mg/mL iodoacetamide, after the IEF.

The Immobiline DryStrip gels were loaded and run on 10% acrylamide isocratic gels using the Ettan DALTsix Electrophoresis System ((GE Healthcare, USA)). Gels were run at 2 W per gel constant power at 20 °C until the bromophenol blue dye front had run off the bottom of the gels [20].

After 2-DIGE, CyDye-labeled proteins were visualized using the Typhoon 9410 imager (GE Healthcare, San Francisco, CA, USA). Gels were scanned at a 100 μm resolution. Images were cropped using ImageQuant™ Version 5.0 (GE Healthcare, USA) to remove areas extraneous to the gel image.

Gel images were analyzed using the Software DeCyder V7.0 (GE Healthcare, USA). All sampled gel image pairs were processed by the DeCyder DIA (Differential In-gel Analysis) software module in order to co-detect and differentially quantitate protein spots in the images. Gel-to-gel matching of the standard spot maps from each gel, followed by statistical analysis of protein abundance change between samples, was done by use the DeCyder BVA (Biological Variation Analysis) software module. Unpaired *t*-test was used to compare protein levels in each group. A fold change of at least 1.5 and a two sided *p*-value < 0.05 was considered statistically significant.

Proteins differentially expressed were selected for mass spectrometry identification. To avoid spot mismatch, protein identification was directly performed on analytic gels.

Fifty-three spots of interest were manually excised from the gels, trypsin digested, and subjected to tandem mass spectrometry analysis.

### 2.7. Nanoscale LC-MS/MS Analysis

LC-MS/MS analysis was carried out using an Easy LC 1000 nanoscale liquid chromatography (nanoLC) system (Thermo Fisher Scientific, Odense, Denmark). The analytical nano LC column was a pulled fused silica capillary, 75 μm in-house packed to a length of 10 cm with 3 μm C18 silica particles from Dr. Maisch (Entringen, Germany).

The peptide mixtures were injected at 500 nL/min directly onto the analytical column. Peptides were eluted using a binary gradient. Mobile phase A was 0.1% formic acid, 2% acetonitrile, while mobile phase B was 0.1% formic acid, 80% acetonitrile. Gradient elution was done at 350 nL/min flow rate, and ramped from 0% B to 30% B in 15 min, and from 30% B to 100% B in additional 5 min; after 5 min at 100% B, the column was re-equilibrated at 0% B for 10 min before the subsequent injection. MS detection was done on a quadrupole-orbitrap mass spectrometer Q-Exactive (Thermo Fisher Scientific, Bremen, Germany) operating in positive ion mode, with nano electrospray (nESI) potential at 1800 V applied on the column front-end via a tee piece. Data-dependent acquisition was done using a top-5 method with resolution (FWHM), AGC target and maximum injection time (ms) for full MS and MS/MS of, respectively, 70,000/17,500, 1 × 10^−6^/5 × 10^−5^, 50/400. Mass window for precursor ion isolation was 2.0 *m*/*z*, while normalized collision energy was 30. Ion threshold for triggering MS/MS events was 2 × 10^−4^. Dynamic exclusion was 15 s.

Data analysis was performed using Proteome Discoverer 1.3 (Thermo Fisher Scientific, Bremen, Germany), using Sequest as search engine, and the HUMAN-refprot-isoforms.fasta as sequence database. Parameters applied for the analysis were the following: MS tolerance 15 ppm; MS/MS tolerance 0.02 Da; fixed modifications: carbamidomethylation of cysteine; variable modification: oxidation of methionine, phosphorylation of serine, threonine and tyrosine; enzyme trypsin; max. missed cleavages 2; taxonomy Human.

We accept only proteins identification performed with two successful peptide identifications (Xcorr >2.0 for doubly charged peptides, >2.5 for triply charged peptides, and >3.0 for peptides having a charge state > 3).

### 2.8. Western Blot Analysis

For 1D Western blot analysis, fifty µg of proteins sample was resolved by pre cast SDS-PAGE (Any kD™ and 4–20% Mini-PROTEAN Precast Protein Gels, Biorad) and electrotransferred to a nitrocellulose membrane with a Trans-blot turbo system (Biorad). Membranes were incubated using the following primary antibodies: HSP60 (1:1000, 4B9/89 Mouse, Thermo scientific, (Bremen, Germany); Cytc (1:1000, D18C7 Rabbit, Cell Signaling, Danvers, MA, USA); HIF-1α (1:1000, D5F3M Mouse, Cell Signaling, Danvers, MA, USA); H3 (1:1000, ,9715, Rabbit, Cell Signaling, Danvers, MA, USA); α-Tubulin (1:1000, 2144, Rabbit, Cell Signaling, Danvers, MA, USA); BRCA1 (1 μg/mL, clone D-20, Santa Cruz, Dallas, TX, USA ).

The detection of a primary antibody was done with anti-mouse horseradish peroxidase-conjugate secondary antibodies (Cell Signaling) for mouse primary antibody, antirabbit horseradish peroxidase-conjugate secondary antibodies (Cell Signaling) for Rabbit primary antibody. Blots were developed using the SuperSignal West Femto ECL substrate (Pierce, Thermo Fisher Scientific Inc., Bremen, Germany). Densitometric software (Alliance 2.7 1D fully automated software) was used to determine the percent distribution of blotted proteins after image acquisition by Alliance 2.7 (UVITEC, Eppendorf, Milan, Italy).

Data were analyzed and plotted using Excel spreadsheet (Microsoft, Redmond, WA, USA), and expressed as mean ± SEM (*N*), where SEM represents the standard error of the mean and *N* indicates the number of experimental repeats. Unpaired *t*-test was used to compare protein levels in each data set. A two sided *p*-value < 0.05 was considered statistically significant.

### 2.9. Pathway Analysis

Proteins differentially expressed were functionally correlated using the software Ingenuity Pathway analysis (Ingenuity Systems, version 42012434, Qiagen, Hilden, Germany, www.ingenuity.com) (IPA). The software builds hypothetical protein interaction clusters based on a constantly updated Ingenuity Pathways Knowledge Base. Data sets containing proteins identifiers of molecules and corresponding expression values were uploaded into the application.

The system algorithmically generated a list of Networks based on their connectivity. Networks were “named” on the most common functional group(s) [21,22]. Each analysis was statistically evaluated by the Fischer exact test. This was used to calculate a *p*-value defining the probability that each biological function and/or disease assigned to that network is due to a random event.

Molecular activity prediction (MAP) function was used to predict the upstream or downstream effects of deregulated protein on specific targets.

### 2.10. Immunofluorescence Microscopy Analysis

Cells (5 × 104/well) were plated onto glass cover slips in the wells of a 6-well culture dish. After the cultures reached 50% confluence, MitoTracker Green (Thermo Scientific) was added to cells and incubated for 1 h at 37 °C. Cells were washed 3× with pre-warmed PBS. Cells were fixed with 4% paraformaldehyde (Sigma-Aldrich) for 30 min. After permeabilization with 0.3% Triton X-100 (Sigma-Aldrich) in phosphate buffered saline (PBS) for 15 min, the cells were blocked with 10% FBS (Biowest, Nuaillé, France)) and 0.1% Triton X-100 in PBS for 1 h at room temperature and then incubated with anti HIF-1α (1:800, D5F3M, Cell Signaling) primary antibodies diluted in PBS containing 3% FBS. Goat anti-mouse Alexa-Fluor-647 (A-21235, Life Technologies) was used as the secondary antibodies at a concentration of 2 µg/mL in PBS containing 1% FBS for 45 min at room temperature. Nuclei were stained with DAPI (4′,6-diamidino-2-phenylindole). Slides were mounted with fluorescent mounting medium (Dako Cytomation) and images were acquired with DMi8 Leica microscope (Leica Microsystems Srl, Milan, Italy). The assays were repeated in three independent biological replicates.

To quantitatively determine subcellular localization of the expressed HIF-1α protein, the relative staining intensities in the nucleus and in the mitochondria were analyzed. Image analysis was performed using ImageJ software (Wayne Rasband, National Institute of Mental Health, Bethesda, MD, USA). In particular, the Coloc2 plugin was used to identify the localization of HIF-1α in the mitochondria (gray regions) and represented in the figure as Overlap Red: Green. Mitotracker and DAPI staining were used to define the mitochondrial and nuclear regions of interest (ROIs) drawn manually. After brightness and contrast adjustment, images were randomly selected from more than 25 cells per cell line. At least 5 ROIs containing non-specific staining were manually selected from the image as background which intensity value was subtracted from the image content. CTFC (corrected total cell fluorescence) was calculated, with the following formula, Integrated Density—(Area of selected cell * Mean fluorescence of background readings). The average ratio between the intensity of fluorescence in the nucleus and in the mitochondria was plotted.

## 3. Results

### 3.1. Mitochondrial Fraction Isolation

**Figure 1:**
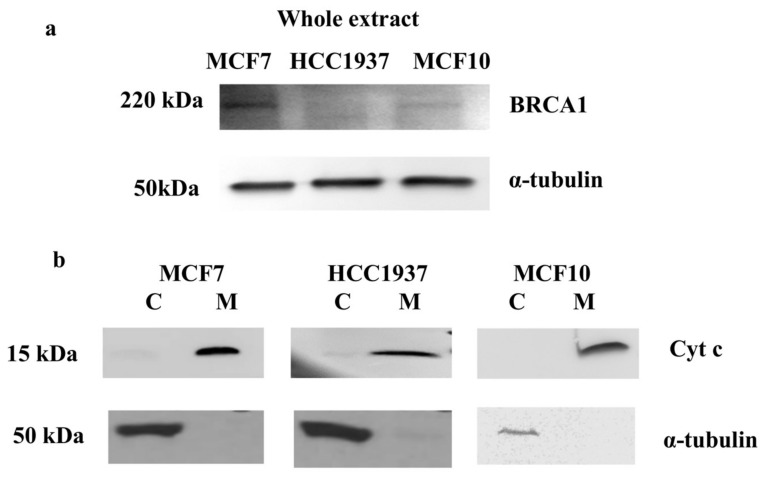
(**a**) BRCA1 western blot analysis. The expression of BRCA1 was assessed by western blot analysis on MCF7, HCC1937 breast cancer cells and MCF10 normal immortalized breast cells. 80 µg of proteins for each cell line were resolved on 4–20% precast polyacrylamide gels (Biorad), electrotransferred to a nitrocellulose membrane with a Trans-blot turbo system (Biorad) followed by immunoblotting. Rabbit monoclonal antibody against BRCA1 (clone D-20, Santa Cruz) was used at a final concentration of 1 μg/mL. HRP-conjugated γ-Tubulin (clone C-20, Santa Cruz) was used at a final concentration of 1μg/mL to ensure equal amount of protein loading. Images were acquired using the Alliance 2.7 system (UVITEC, Eppendorf, Milan, Italy). (**b**) Mitochondria isolation. Proteins extracts from mitochondrial (M) and cytosolic (C) fractions were analyzed by western blot analysis. The upper blots are representative of cyt c expression in breast cancer cells (MCF7 and HCC1937) as well as in normal immortalized cells (MCF10). The lower blots show α-tubulin expression in breast cancer cells (MCF7 and HCC1937) as well as in normal MCF10 cells. 50 µg of proteins were resolved by SDS-PAGE using Any kD™ Mini-PROTEAN precast gels and electrotransferred to a nitrocellulose membrane with a Trans-blot turbo system (Biorad). Images were acquired using the Alliance 2.7 system (UVITEC, Eppendorf, Milan, Italy).

The experimental work flow is based on the analysis of breast cancer cells expressing or not *BRCA1*, the endogenous level of BRCA1 protein in each analyzed cells is shown in Figure 1a.

The efficiency of the mitochondrial isolation method was evaluated by western blot analysis. In Figure 1b is shown the western blot of cytochrome c (cyt c) performed on protein extracts from mitochondrial and cytosolic fraction. The high levels of cyt c in the mitochondrial fractions of all analyzed cells, clearly confirm that the method is able to enrich the fractions of interest.

### 3.2. 2D DIGE and Mass Spectrometry Identifications

**Figure 2:**
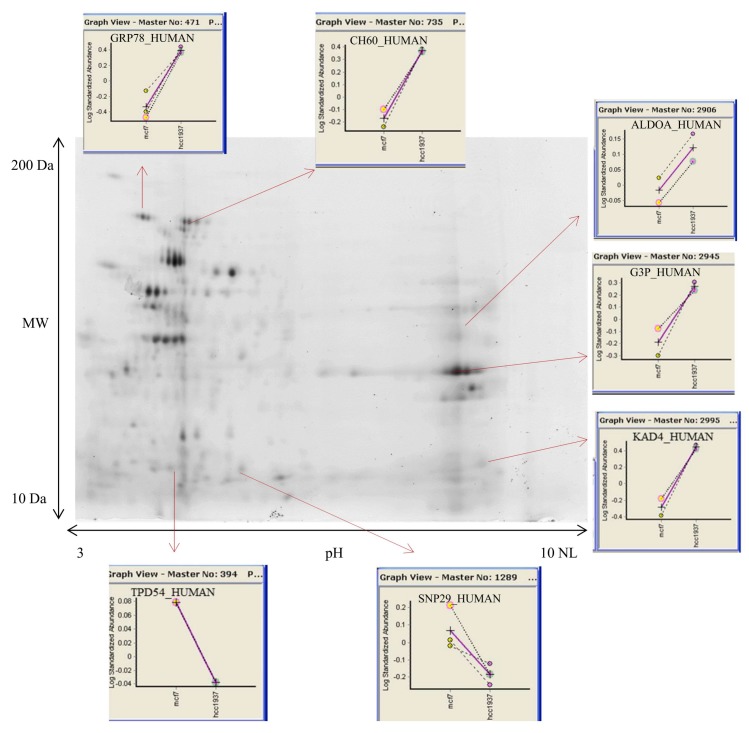
Representative image of mitochondrial 2D DIGE profile. Mitochondria from breast cancer cells were isolated and analyzed by DIGE analysis. Isoelectrofocusing was carried out on 3–10 NL IPGstrip, 24 cm length. Second dimension was performed on 10% SDS-PAGE. Gel images were analyzed using Decider software.

To address the role of mitochondria in the *BRCA1*-mediated metabolic rewiring of breast cancer, we compared the mitochondrial proteome of sporadic breast cancer cells (MCF7) with hereditary breast cancer cells (HCC1937). The analysis was done by 2D-DIGE approach coupled with LC-MS/MS characterization. The representative 2D maps separations are shown in Figure 2. Gels image were analyzed using the module DIA and BVA of software Decyder.

The analysis was designed to obtain protein quantitation within replicate gels in both cancer models as well as across the two groups.

First, the gels were analyzed with the DIA module. For the detection, we used the 6.0 algorithm. The estimated number of spots for each co-detection procedure was set to 2500.

All the gel images generated by the DIA were matched later in the DeCyder BVA (Biological Variation Analysis) software module. We selected fifty-three spots with a fold change of at least 1.5 (*p*-value < 0.05).

Proteins identifications with relative quantitation are summarized in Supplementary file 1. In the table are reported: spot numbers, protein identifier, gene mane, number of unique peptides, score of identifications and fold change of proteins spot in HCC1937 cancer cells versus MCF7 cancer cells. Moreover, in the table is also reported the known cellular localization of the identified proteins. This information was obtained by comparing our proteins data set with MitoCarta2.0, an updated inventory of mammalian mitochondrial proteins. Thirty-three proteins have been reported to have a mitochondrial localization, seven were categorized as “possible mitochondrial proteins” and fourteen were not mitochondrial proteins [23,24]. 

Among the differentially expressed proteins, the 60 kDa heat shock protein, the adenylate kinase A4 as well as the glyceraldehyde-3-phosphate dehydrogenase and the Fructose-bisphosphate aldolase A, were significantly up regulated in the hereditary breast cancer cells compared to the sporadic model.

### 3.3. Assessment of the Mitochondrial Localization of HSP60

Western blot analysis of cytosolic and mitochondrial fractions of both cancer cells was done in order to evaluate and to confirm the peculiar localization of the HSP60 in the mitochondria of HCC1937 cells.

**Figure 3:**
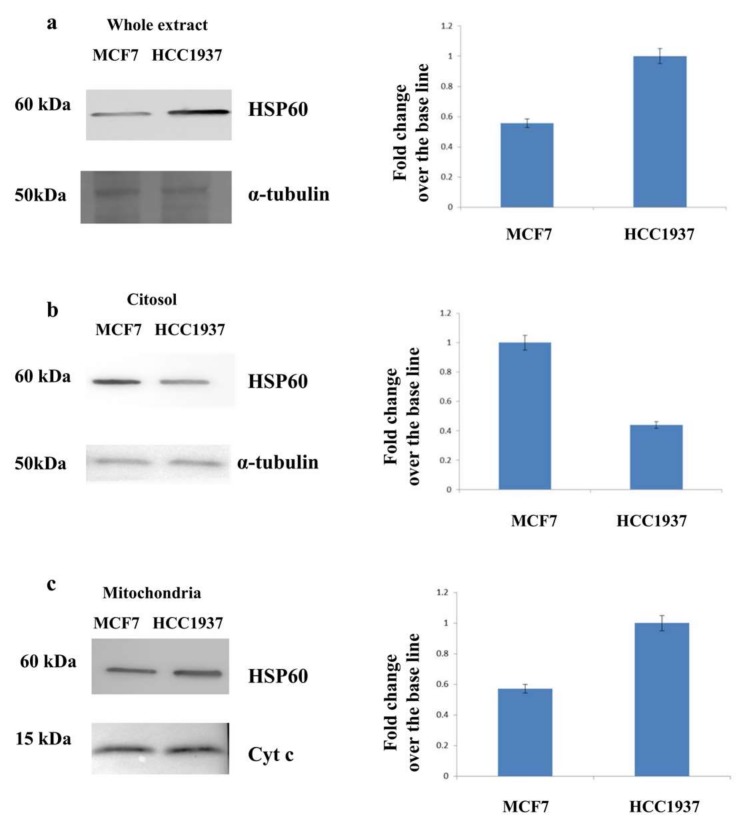
Western blot analysis of Cytosolic and mitochondrial fractions of HCC1937 and MCF7 cancer cells. Left panel: (**a**) HSP60 resulted upregulated in the whole extract of hereditary breast cancer cells (HCC1937) compared to sporadic breast cancer cells (MCF7); Western blot signals were normalized using α-tubulin as housekeeping proteins. (**b**) HSP60 was down-expressed in the cytosolic fraction of hereditary cancer cells (HCC1937) compared to sporadic breast cancer cells (MCF7); Western blot signals were normalized using α-tubulin as housekeeping proteins. (**c**) HSP60 was upregulated in the mitochondrial fraction of hereditary breast cancer cells (HCC1937) compared to sporadic breast cancer cells (MCF7); Western blot signals were normalized using cyt c as housekeeping proteins. Right panel: Densitometry analysis for each analyzed proteins. Analysis was performed using three independent experiments. Data are mean ± SEM (*N* = 3) *p* < 0.05. For each western blot 50 µg of proteins were resolved by SDS-PAGE using Any kD™ Mini-PROTEAN precast gels and electrotransferred to a nitrocellulose membrane with a Trans-blot turbo system (Biorad). Images were acquired using the Alliance 2.7 system (UVITEC, Eppendorf, Milan, Italy) and analyzed by excel spreadsheet.

As shown in Figure 3 panel a, the HSP60 was specifically up regulated in the whole extract of hereditary cancer cells compared to sporadic cells. The in depth analysis of cytosolic and mitochondrial fraction (Figure 3 panel b and c) reveals that the levels of HSP60 are much higher in the mitochondria of cells carrying a mutation on *BRCA1* gene.

### 3.4. IPA Analysis

**Figure 4:**
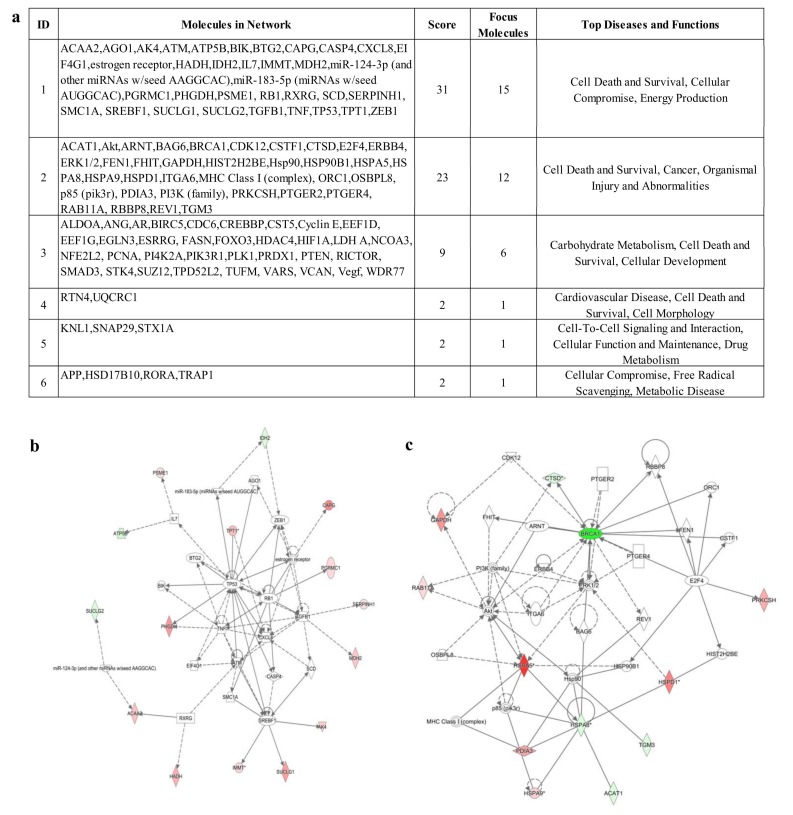
IPA analysis. (**a**) List of networks generated using DIGE quantitative data and mass spectrometry identification summarized in Table S1. (**b**) Top network 1. (**c**) Top network 2. In green boxes are indicated proteins that resulted decreased in HCC1937 vs. MCF7 breast cancer cells. In red boxes are indicated proteins which levels resulted increased in HCC1937 vs. MCF7 breast cancer cells.

The analysis of differentially expressed proteins allowed to map the fifty-three molecules in six networks (Figure 4 panel a).

The most relevant networks had functions associated with cell death and survival, cellular compromise, energy production and organismal injury and abnormalities (Figure 4 panel b and c). 

The central hubs of several networks are key molecules of carcinogenesis process such us Akt, P53, and HIF-1α [25,26,27].

**Figure 5:**
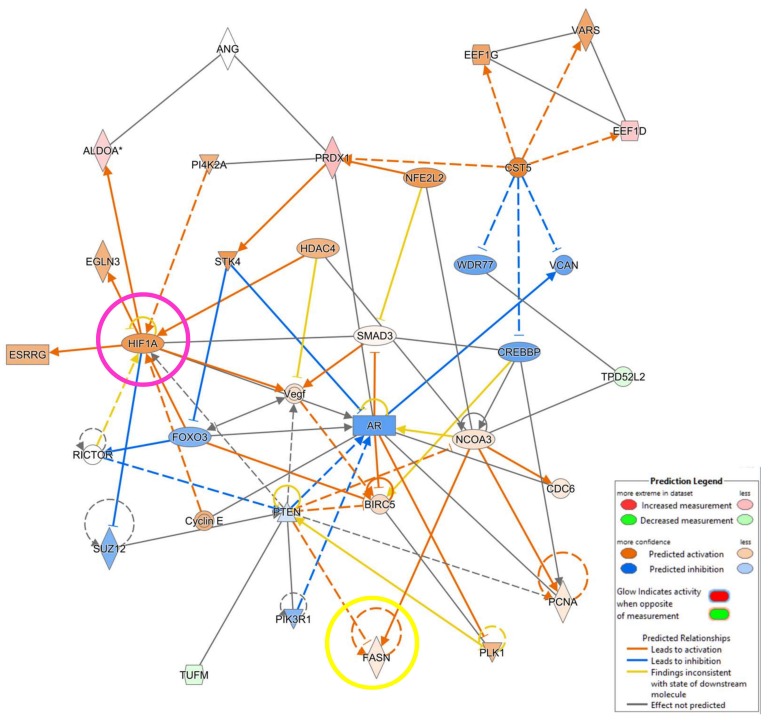
IPA analysis: Molecular activity predictor (MAP). Top network 3. MAP function by integrating DIGE quantitative data with IPA knowledge database predict the specific overexpression of FASN (yellow circle) as well as the upregulation of HIF-1α (pink circle).

In order to further investigate the relationships between deregulated molecules and central hub included in the networks, we used the Molecule Activity Predictor (MAP), a function of IPA that anticipates the upstream/downstream effects of activation or inhibition of molecules included in the analyzed dataset. Using this tool, we established the in-silico up regulation of Fatty acid synthase (FASN), a key enzyme underlying fatty acid synthesis, as well as the over expression of HIF-1α, a crucial regulator of the hypoxia response (Figure 5).

The expression of HIF-1α together with the peculiar subcellular localization was further explored by western blot analysis.

### 3.5. Validation of the Expression of HIF-1α by Western Blot Analysis

**Figure 6:**
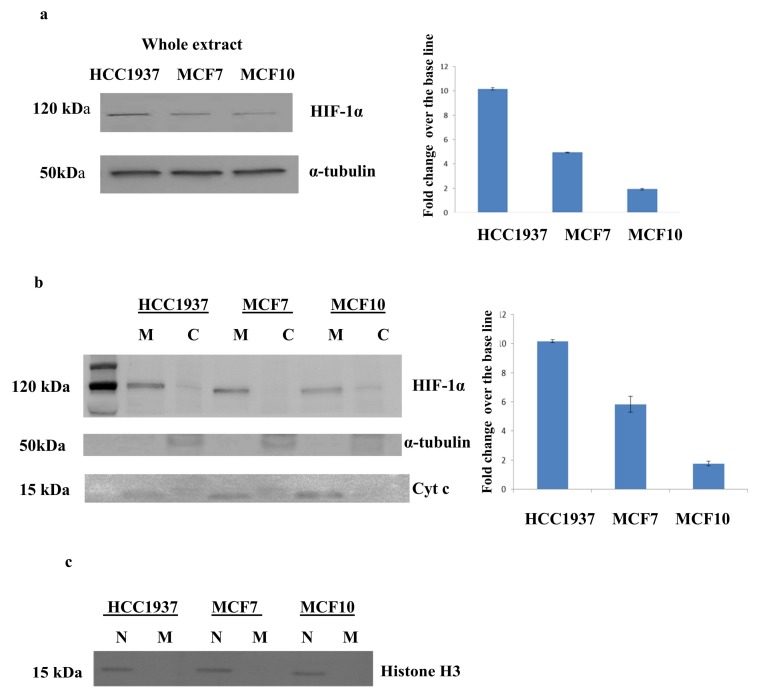
Mitochondrial localization of HIF-1α. Western blot analysis of Cytosolic and mitochondrial fractions of HCC1937 and MCF7 cancer cells. Left panel: (**a**) HIF-1α was upregulated in the whole extract of hereditary breast cancer cells (HCC1937) compared to sporadic breast cancer cells (MCF7); Western blot signals were normalized using α-tubulin as housekeeping proteins. (**b**) HIF-1α was upregulated in the mitochondrial fraction of hereditary breast cancer cells (HCC1937) compared to sporadic breast cancer cells (MCF7); Western blot signals were normalized using cyt c as housekeeping proteins. Right panel: Densitometry analysis for each analyzed proteins. Analysis was performed using three independent experiments. Data are mean ± SEM (*N* = 3) *p* < 0.05. (**c**) Western blot showing the expression of the nuclear marker Histone H3 on mitochondrial and nuclear extract of HCC1937 breast cancer cells, MCF7 breast cancer cells and MCF10 normal immortalized breast cells.

The cellular localization of HIF-1α was investigated by western blot analysis, as shown in Figure 6 panel a. The level of HIF-1α is much higher in the whole extract of both cancer cell lines compared to immortalized normal breast cells. A careful analysis of western blot images unveils that the expression of HIF-1α is significantly increased in the hereditary breast cancer cells, suggesting a specific role of *BRCA1* in regulating this factor.

The specific analysis of subcellular fraction (Figure 6, panel b) reveals that HIF-1α has a clear mitochondrial localization, and that this localization might be dependent from *BRCA1* mutation status. The levels of HIF-1α in the mitochondrial fraction of *BRCA1* mutated breast cancer cells is significantly increased compared to sporadic models. A further experiment on mitochondrial and nuclear extract of breast cells was done to assure that the data reported here were not the result of a cross contamination of nuclei during centrifugation process. As shown in Figure 6, panel c, the nuclear marker Histone H3 is detectable only in the nuclear fractions of analyzed breast cancer cells, confirming that the experiments were correctly performed and that HIF-1α might indeed be inside the mitochondria.

### 3.6. Stable Transfection of BRCA1 in Hereditary Breast Cancer Cell Line (HCC1937)

**Figure 7:**
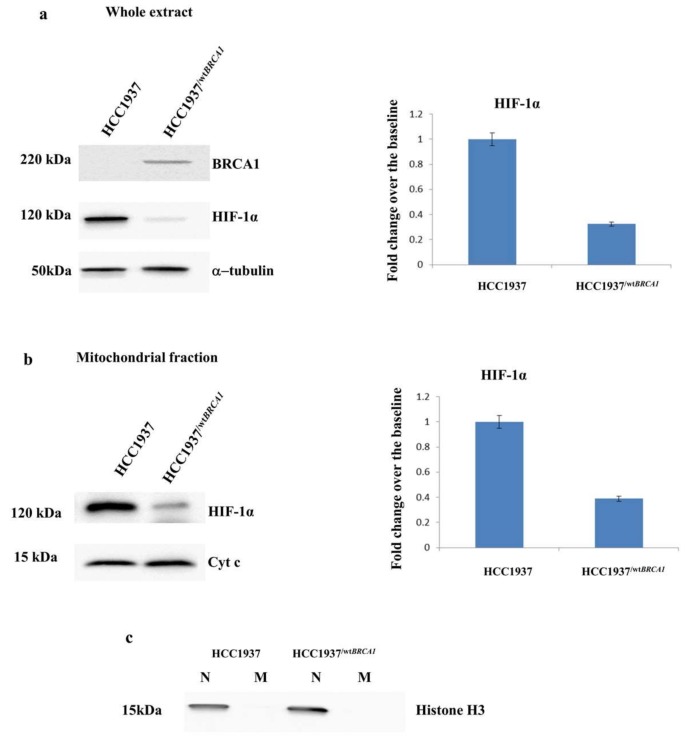
HIF-1α localization in HCC1937 and HCC1937/^*wtBRCA1*^ breast cancer cell lines.Left panels: Western blot analysis of Cytosolic and mitochondrial fractions of HCC1937 and HCC1937/^*wtBRCA1*^ cancer cells. (**a**) HIF-1α was upregulated in the whole extract of hereditary breast cancer cells (HCC1937) compared to HCC1937/^*wtBRCA1*^*.* Western blot signals were normalized using α-tubulin as housekeeping proteins. (**b**) HIF-1α was upregulated in the mitochondrial fraction of hereditary breast cancer cells (HCC1937) compared stable transfected cells (HCC1937/^*wtBRCA1*^*)*; Western blot signals were normalized using cyt c as housekeeping proteins. Right panel: Densitometry analysis for each analyzed proteins. Analysis was performed using data from three independent experiments. Data are mean ± standard error of the mean (SEM) (*N* = 3) *p* < 0.05. (**c**) The nuclear marker Histone H3 on mitochondrial and nuclear extract of analyzed breast cancer cells assesses the purity of mitochondrial fractions.

With the attempt to clearly relate the expression of *BRCA1* and the peculiar subcellular localization of HIF-1α and HSP60, the *BRCA1* full length was transfected in the hereditary breast cancer model. The cell lines HCC1937/*^wtBRCA1^* was produced in our laboratory as previously reported [14,15]. Since *BRCA1* reconstituted HCC1937 cells (HCC1937/*^wtBRCA1^*) differs from parental cell line (HCC1937) only for the expression of *BRCA1*, as demonstrated by western bot analysis (Figure 7a), they can be considered a valuable and reliable model to evaluate the influence of *BRCA1* on the expression and localization of HIF-1α.

The comparison between the whole protein extracts of HCC1937 and HCC1937/^*wtBRCA1*^ allowed us to clearly associate the increase of HIF-1α with the expression of BRCA1 (Figure 7a), accordingly with previously studies [28,29,30,31]. Western blot analysis on mitochondrial fraction confirmed the hypothesis that *BRCA1* might influence the mitochondrial localization of HIF-1α (Figure 7b).

**Figure 8:**
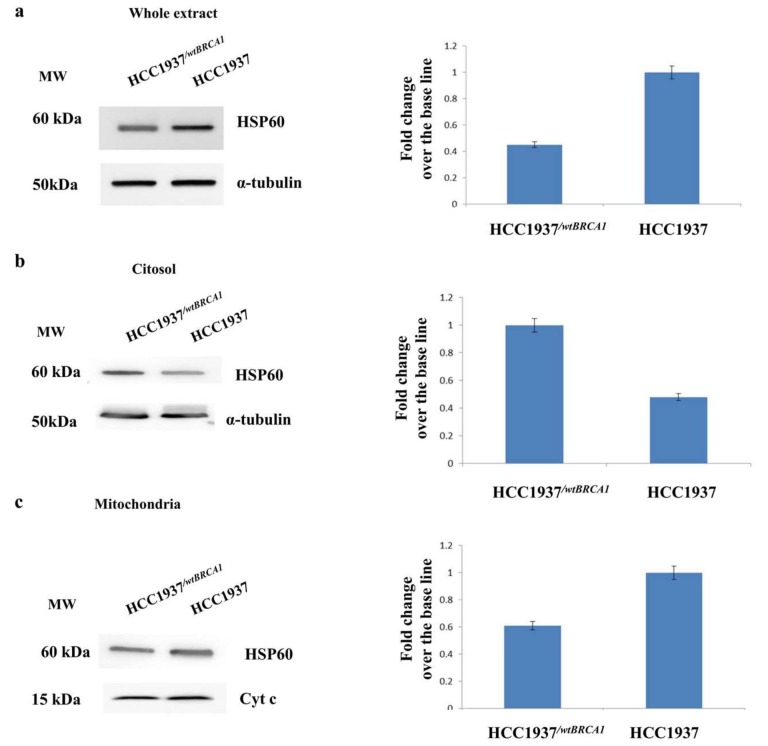
Western blot analysis of Cytosolic and mitochondrial fractions of HCC1937 and HCC1937/^*wtBRCA1*^ cancer cells. Left panel: (**a**) HSP60 resulted upregulated in the whole extract of hereditary breast cancer cells (HCC1937) compared to *BRCA1* transfected cancer cells (HCC1937/^*wtBRCA1*^); Western blot signals were normalized using α-tubulin as housekeeping proteins. (**b**) HSP60 was down-expressed in the cytosolic fraction of hereditary cancer cells (HCC1937) compared *BRCA1* transfected cancer cells (HCC1937/^*wtBRCA1*^); Western blot signals were normalized using α-tubulin as housekeeping proteins. (**c**) HSP60 was upregulated in the mitochondrial fraction of hereditary breast cancer cells (HCC1937) compared to *BRCA1* transfected cancer cells (HCC1937/^*wtBRCA1*^); Western blot signals were normalized using cyt c as housekeeping proteins. Right panel: Densitometry analysis for each analyzed proteins. Analysis was performed using three independent experiments. Data are mean ± SEM (*N* = 3) *p* < 0.05. For each western blot 50 µg of proteins were resolved by SDS-PAGE using Any kD™ Mini-PROTEAN precast gels and electrotransferred to a nitrocellulose membrane with a Trans-blot turbo system (Biorad). Images were acquired using the Alliance 2.7 system (UVITEC, Eppendorf, Milan, Italy) and analyzed by excel spreadsheet.

Similarly to HIF-1α, the subcellular localization of HSP60 was evaluated in the HCC1937/^*wtBRCA1*^ compared to HCC1937. Again, western blot data confirmed that the expression of BRCA1 might change the cellular distribution of HSP60 (Figure 8)

### 3.7. Immunofluorescence Analysis on HIF-1α

**Figure 9:**
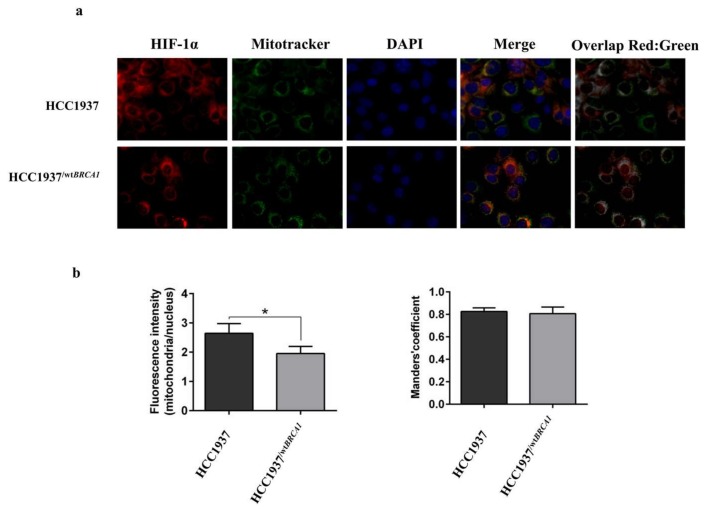
HIF-1α sub-cellular localization by fluorescence microscopy. (**a**) Representative images of HIF-1α (red), Mitotracker (green), nuclei stained with DAPI (Blue). The overlap of the three channels is represented by the merge, while the overlay of the red and the green identifies gray regions of the colocalization of HIF-1α and the Mitotracker. Scale bar, 50 μm. (**b**) The ratio of the HIF-1α fluorescence intensity in the mitochondria and in the nucleus is reported in the histogram as mean ± standard error of the mean (SEM). Significant differences between HCC1937 and HCC1937/^*wtBRCA1*^ were determined by Student’s *t* test, * *p* < 0.05.

The most intriguing finding, according to our opinion, is the mitochondrial localization of HIF-1α, that to our knowledge, it has been reported only once in colon cancer cells [32]. To further confirm our finding in breast cancer cells, we provide here microscopy fluorescence data. As shown in Figure 9a, about 80% of cytoplasmic HIF-1αlocalizes in the mitochondria, while 20% of the signal is exclusive of the cytoplasm. The mitochondria/nucleus ratio of fluorescence intensity is higher in the HCC1937 line compared to HCC1937/^*wtBRCA1*^ lines. The ratio is about 2.87, and 1.91, respectively, in HCC1937 and HCC1937/^*wtBRCA1*^ (Figure 9b)*,* suggesting that the amount of HIF-1α moving into the mitochondria is dependent from BRCA1 expression.

## 4. Discussion

Recently, “mitochondrial medicine” has turned up as one of the main topics in a number of papers, some of which are focused on classical mitochondrial diseases, while many other deal with diseases in which mitochondrial dysfunction and/or mitochondrial stress signaling play important roles. 

The increased interest of researchers in the biology of mitochondria and the discovery of their involvement in many types of diseases and ageing has been the driving force of mitochondrial proteomics. Mitochondrial proteomics aims to study changes in the levels of mitochondrial proteins as well as to characterize proteins that should have dual localization, and/or proteins that only in specific physiological/pathological conditions associate with mitochondria or are imported into mitochondria.

Here, 2D-DIGE was coupled with mass spectrometry, bioinformatics and molecular biology to elucidate the role of mitochondria in *BRCA1*-driven tumorigenesis. The study of the compartmentalization of proteins is essential for the complete understanding of their function and to elucidate the pathways in which they are involved.

As first result we propose the peculiar localization of HSP60 protein in response to *BRCA1* mutation status. In physiological conditions, HSP60 is a protein of the mitochondrial matrix that plays an important role in facilitating protein folding and in the maintenance of mitochondrial integrity. HSP60 associates with caspase-3 and it is responsible for the induction of its maturation and activation. The association of HSP60 with procaspase-3 and the 30-kDa caspase-3 occurs only in the cytosol [33].

When HSP60 is released into cytosol, it plays a pro-apoptotic role; conversely, when HSP60 is accumulated into mitochondria, it plays a pro survival role. The accumulation of HSP60 into mitochondria, that we observe in our model, is consistent with a pro-survival role of HSP60 and may help to elucidate the pathways that lead to apoptosis suppression in *BRCA1* mutated breast cancer cells.

As second result, we found that the expression of many factors associated with the metabolic rewiring of breast cancer cells is coherent with the switch versus the anaerobic glycolysis.

Mitochondrial energy production is the more efficient method for energy production. In cancer cells, when the level of oxygen is low, pyruvate is prevalently converted to lactate instead of acetyl CoA; hypoxia induces the expression of HIF-1α which, in turn, regulates the expression of several genes, some of which are involved in the glycolytic pathway [34,35,36]. HIF-1α is able to block CoA metabolism in mitochondria as well as mitochondrial biogenesis and oxygen consumption. In *BRCA1* mutated cells line, the high levels of HIF-1α are coherent with the metabolic switch required for cellular adaptation to hypoxia [37].

In highly proliferative cancer cells a de novo synthesis of fatty acids is observed. They are the major components of cell membrane and constitute the key substrates for energy production, lipid modification of proteins, and signaling molecule production. In several types of cancer, lipogenesis is increased by overproduction of fatty acid synthase (*FASN*), the enzyme that converts acetyl CoA and malonyl CoA to fatty acids and acts as an oncogene by promoting cancer cell proliferation and growth [38]. Coherently with this evidence, using the map function and our DIGE quantitative data, we predict the activation of fatty acid synthase in *BRCA1* mutated cell lines.

In *BRCA1* mutated cells, we also observed the up regulation of adenylate kinase A4 (AK4). The oncogenic potential of many cancers has been related with high levels of AK4. AK4 is an important regulator of cellular homeostasis through the regulation of cellular energy charge and AMPK activation. AK4 contributes to cancer process by maintaining the proper balance of adenosine nucleotides and by controlling the bioenergetic program switch of cancer cells [39].

These evidences are in agreement with the switch toward aerobic glycolysis exhibited by *BRCA1* mutated breast cancer cells.

Finally, the up regulation of two key enzymes, the glyceraldehyde-3-phosphate dehydrogenase and the fructose-bisphosphate aldolase A, reported as “possible mitochondrial”, correlate with the up regulation of glycolysis pathway supporting the Warburg’s hypothesis [34].

Using subcellular fractionation analysis on purified mitochondria, we have found that in breast cancer cells the metabolic switch is speeded up in response to the presence of a peculiar mutation on *BRCA1* gene.

## 5. Conclusions

The main finding of this paper is that endogenous HIF-1α might localize with mitochondrial fractions in response to *BRCA1* mutated status. Our hypothesis is strongly supported by the transfection of *BRCA1* full length in HCC1937, which restores the levels of *BRCA1* in these cells.

Although HIF-1α is best known as a key regulator component of hypoxia response, we hypothesize that HIF-1α may have additional functions outside the nucleus. More specifically, it is possible that HIF-1α in the mitochondria might regulate the activity of specific enzymes, or—much more intriguingly—it might direct the expression of mitochondrial DNA acting as transcription factor.

Our results establish a connection between *BRCA1* and mitochondria pathways, capable of triggering metabolic changes which, in turn, sustain the high energetic and anabolic requirements of the malignant phenotype. By controlling HIF-1α, *BRCA1* haploinsufficiency might drive metabolic rewiring in breast epithelial cells, acting as a push toward malignant transformation.

## Supplementary Materials

The following are available online at http://www.mdpi.com/2227-7382/6/2/16/s1, Table S1: Mass spectrometry identifications of differentially expressed proteins.
